# The Relationship Between the COVID-19 Pandemic and Vaccine Hesitancy: A Scoping Review of Literature Until August 2021

**DOI:** 10.3389/fpubh.2021.747787

**Published:** 2021-09-28

**Authors:** Matilde de Albuquerque Veloso Machado, Bjelle Roberts, Brian Li Han Wong, Robin van Kessel, Elias Mossialos

**Affiliations:** ^1^Department of International Health, Care and Public Health Research Institute (CAPHRI), Maastricht University, Maastricht, Netherlands; ^2^Merck Sharp & Dohme (MSD), Brussels, Belgium; ^3^Medical Research Council Unit for Lifelong Health and Ageing (MRC), London, United Kingdom; ^4^Association of Schools of Public Health in the European Region (ASPHER), Brussels, Belgium; ^5^Studio Europa, Maastricht University, Maastricht, Netherlands; ^6^Research Committee, Global Health Workforce Network (GHWN) Youth Hub, World Health Organization, Geneva, Switzerland; ^7^Department of Health Policy, London School of Economics and Political Science, London, United Kingdom; ^8^Institute of Global Health Innovation, Imperial College London, London, United Kingdom

**Keywords:** COVID-19, immunisation, media, vaccines, vaccine hesistancy, vaccine uptake, vaccine confidence

## Abstract

**Background:** Vaccines have been contributing to eradicate or drastically reduce the incidence of common diseases. Simultaneously, vaccine hesitancy is considered among the top ten global health threats. The COVID-19 pandemic has caused a tremendous impact on health, economics, and society worldwide, while also reinforcing faulty beliefs about the necessity of vaccine programs as a whole. This study aims to synthesise evidence on the impact of the COVID-19 pandemic on vaccine hesitancy.

**Methods:** A scoping review of literature between 1 January 2020 and 1 August 2021 was performed.

**Results:** COVID-19 vaccine acceptance decreased from more than 70 to <50% in 8 months starting from January 2020. Healthcare professionals demonstrate higher rates of vaccine receptivity than the public, which was more influenced by (social) media. The circulation of misinformation was associated with increased fear of side effects related to COVID-19 vaccines. Regarding other vaccines coverage, parents' intentions to vaccinate their children against influenza increased 15.8% during the COVID-19 pandemic so far. Nonetheless, the number of vaccines administered decreased, influenced by factors like fear of being exposed to the virus at healthcare facilities and restrictions.

**Conclusions:** Several efforts should be undertaken to improve vaccine acceptance and coverage now and beyond the pandemic to optimal population protection.

## Introduction

**Figure 1:**
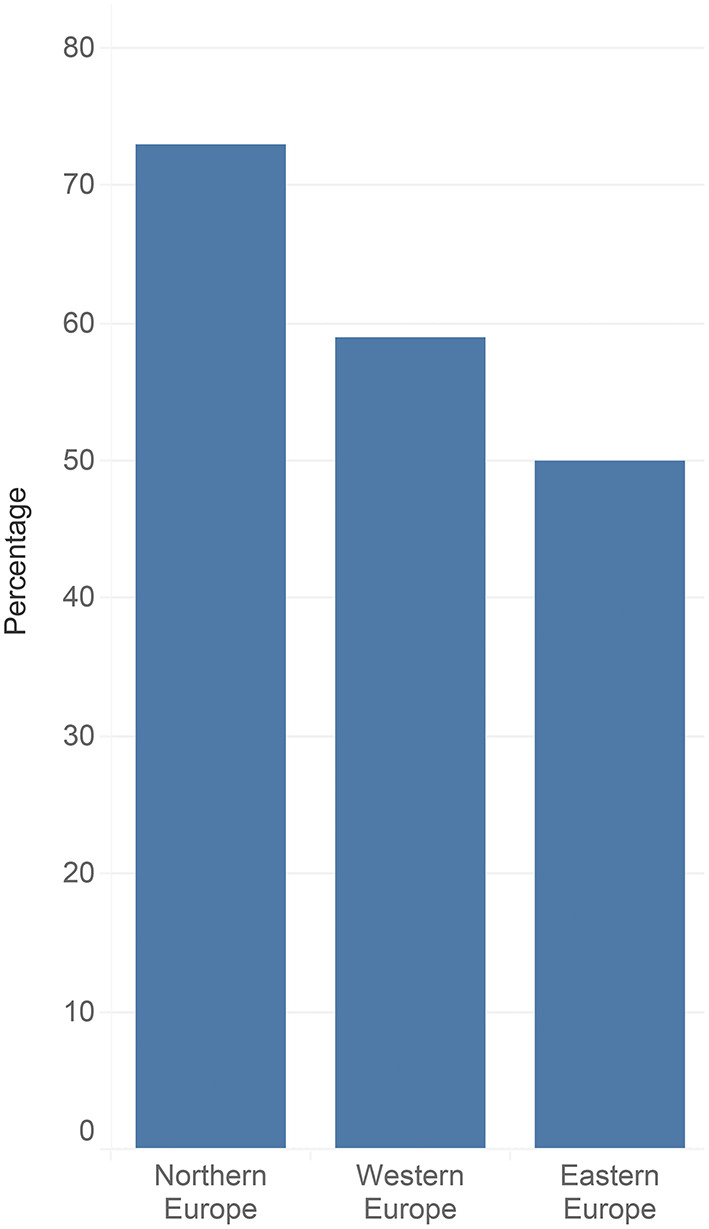
Vaccine hesitancy rates across European regions (4).

Vaccines are “one of the most successful and cost-effective interventions to improve health outcomes” [(1), p. 3]. Historically, vaccines have been essential to eradicating or drastic reduction in the incidence of common diseases (2), saving countless lives and improving population health and well-being globally (1). However, sufficient vaccination coverage rates are essential to preventing excess mortality and potential complications caused by vaccine-preventable diseases (1). Based on a Wellcome Trust survey containing data from 140.000 respondents worldwide, 79% of people regard vaccines as safe (3). However, vaccine acceptability and confidence are lower in high-income countries (see Figure 1).

The WHO defines vaccine hesitancy as “the reluctance or refusal to vaccinate despite the availability of vaccines” [(1), p. 3]. Vaccine hesitancy is a wicked problem, given its innumerable causes, difficulty to describe, and having no clear solution [(5), p. 1]. It is context-specific, influenced by different social motivations, contexts and reasons that define individuals' intentions to vaccinate. The WHO model of vaccine hesitancy includes three factors: (1) confidence (e.g., the extent to which a vaccine or provider is trusted); (2) complacency (i.e., a situation wherein an individual does not perceive the need for and the value of vaccines); and (3) convenience (e.g., barriers to access for vaccination services) (1). In 2019, the WHO named vaccine hesitancy as one of the top ten global health threats, declaring that it threatens the progress of addressing vaccine-preventable diseases (2). There are different attitudes, beliefs and behaviours toward vaccines; thus, vaccine-hesitant individuals represent a heterogeneous group. Some individuals accept vaccines, some remain concerned about them, some may refuse or delay specific vaccines but accept others, and some may refuse all vaccines (5). Although parents receive advice from doctors and public health authorities to vaccinate their children, many parents still express concerns about vaccine safety (4). Therefore, it is difficult to determine how to address vaccine hesitancy (5). In high-income countries, anti-vax communities that mobilise opposition to vaccines are the predominant cause for vaccine hesitancy, chiefly concerning vaccine safety (4).

Concerns about vaccines are not new. In the United Kingdom (UK), in 1853, smallpox vaccination was made mandatory by law, culminating in widespread vaccine resistance through Anti-Vaccination Leagues. In 1898, the enforcement was relaxed, and parents were allowed to opt-out of vaccinating their children. A (now retracted) 1998 Lancet article links the MMR vaccine and autism; this remains a notable example of unfounded safety concerns about vaccines. While retracted and rebuked, the article continues to influence vaccine safety concerns. These ideas caused an enormous impact on public health, with the decrease of MMR vaccination rates from 92% (1995) to 79% (2003) in the UK (4). Nowadays, non-factual information about vaccines can spread rapidly through social media platforms, promoting a culture of distrust in vaccinations (5) and fear about vaccine safety and its side effects (4).

The coronavirus disease 2019 (COVID-19) pandemic has caused millions of infections and hundreds of thousands of deaths worldwide (6). Therefore, a vaccine to prevent COVID-19 infection is vital for tackling the pandemic (7). However, next to an equitable distribution of vaccines across countries [see Papadakis and Spernovasilis (8–10)], high rates of vaccine acceptance and coverage are crucial for the success of immunisation programs. The purpose of this scoping review is to review the impact of the COVID-19 pandemic on vaccine hesitancy globally, summarising the trends on COVID-19 vaccine acceptance, factors that primarily influence vaccination acceptance in the COVID-19 era, and how other vaccination programs have been affected by the pandemic. Methodological details and limitations are included in the Supplementary Material. In short, we set out to explore the question: *How has the COVID-19 pandemic been influencing vaccine hesitancy?* The thematic groups used for this review and which formed the units of analysis were:

1. Trends in COVID-19 vaccine hesitancy
2. Factors that influence vaccine acceptance in the COVID-19 era
3. Impacts of the COVID-19 pandemic on the uptake of other vaccines.

## Trends in COVID-19 Vaccine Hesitancy

COVID-19 vaccine acceptance has been substantially influenced by diverse factors, such as location, socioeconomic status, occupation, beliefs, and safety and efficacy concerns. Lin et al. (11) presented that a decline in general COVID-19 vaccine receptivity (irrespective of the manufacturer of the vaccine) was noticed from March 2020 (>70%) to October 2020 (<50%). The countries most receptive to COVID-19 vaccines at that time were Brazil, South Africa, Denmark and the UK, with ~80% of acceptance. Conversely, countries such as Russia and France presented a considerably high rate of uncertainty related to COVID-19 vaccines, with a vaccine acceptance of about 55%. The predominant factors influencing the acceptance of these new vaccines are perceived risk and vaccine safety and effectiveness concerns. Common factors contributing to hesitation toward COVID-19 vaccines included fear of side effects, concerns about vaccine safety, and their effectiveness. Moreover, the belief that vaccines are unnecessary, inadequate information and unknown/limited duration of immunity were associated with low rates of COVID-19 vaccine acceptance (11). Another factor identified by Lin et al. (11) was the expedited vaccine development that brought about distrust and doubt regarding the development, clinical trials and authorisation process of vaccines, leading to increased vaccine safety concerns. Furthermore, conspiracy theories and distrust in government or healthcare professionals contribute to the factors that create more doubts and objections to vaccination (11).

Parents may have different vaccination intentions for their children compared to themselves. Studies are not conclusive about the percentage of parents who are receptive to vaccinating their children with the new vaccines. However, parents who indicate refusal for themselves are unlikely to vaccinate their children (11). A multi-methods approach study in England that included an online cross-sectional survey and semi-structured interviews showed that most survey participants were more likely to accept a COVID-19 vaccine for themselves than for their children (12). The critical reasons for parents not accepting vaccines were accelerated vaccine development and concerns about safety and efficacy from their perspective. Moreover, parents' reasons for lower receptiveness to vaccinate their children were that children are at lower risk of developing severe outcomes from COVID-19 infection compared to adults. Parents emphasised that transparent information surrounding vaccine development, efficacy, and safety must be provided by the national public health authorities to make informed decisions about vaccination (12).

International studies have demonstrated that healthcare professionals reported higher vaccine acceptance rates than the general public, although rates differed depending on the country (11). A cross-sectional online survey conducted in Greece found high acceptance for COVID-19 vaccines (78.5%; again, irrespective of the manufacturer) and 74% of vaccination coverage for the influenza vaccine in healthcare professionals. The absence of fear of vaccine safety and information received from the Greek public health authorities was associated with the likelihood of receiving a COVID-19 vaccine. Amongst healthcare professionals, the primary reasons for refusing vaccination were fear of potential side effects and concerns regarding the rapid development of COVID-19 vaccines (13).

## Factors That Influence Vaccine Acceptance in the COVID-19 Era

Surveys analysed by Marco-Franco et al. (14) demonstrate low levels of people's trust in information spread on media and social networks. However, citizens trust in their capacity to distinguish false news from accurate information. Confusion due to the constant circulation of false news influences and increases the public's fear of side effects related to COVID-19 vaccines, raising additional questions and concerns about the vaccines. In 2018, 80% of respondents of the Eurobarometer questionnaire on false news found false news several times a month or more, and 37% responded that they found it daily or almost every day. In September 2020, survey respondents in the UK had encountered information or news about the COVID-19 that they thought was false or misleading, and a significant percentage of them received false news a few times a week (14).

On 15 March 2021, various European countries suspended the Oxford/AstraZeneca vaccine, supported by deaths arising from blood clots (15, 16). Recent studies found that—in the UK and Denmark—the intentions and attitudes toward COVID-19 vaccines did not change despite the negative news surrounding AstraZeneca (17, 18). Simultaneously, different studies show a decrease in vaccine acceptance, either directly through surveys in Saudi Arabia (19) or indirectly through reduced turnout for scheduled vaccinations in Ecuador (although the exact reason for not showing for the vaccinations remains unclear) (20).

Non-factual and misleading information about COVID-19 vaccination and pervasive anti-vaccine content continue to proliferate on social media platforms (21). Wilson et al. (22) examined the global effects of social media on vaccine hesitancy and found that over time, the prevalence of foreign disinformation negatively influences vaccination coverage and increases the likelihood of negative discussions on vaccines surfacing on social media. Furthermore, at a national level, studies have shown that the use of social media to organise offline action greatly influences beliefs that vaccines are unsafe (22).

YouTube is also a source of false news and non-factual information. Basch et al. examined 87 videos in 2017 using “vaccine safety” and “vaccines and children” and discovered that 65% of them indicate anti-vaccine positions. Puri et al. (21) state that, amongst the top YouTube videos, were identified videos containing non-factual information when searching terms as “COVID-19” and “coronavirus.” Those videos had until then over 60 million views. Social media content has been focused on the rapid spread of COVID-19 information and the resultant global pandemic (21).

## Impacts of the COVID-19 Pandemic on the Uptake of Other Vaccines

Google searches on anti-vaccine terms have increased during the COVID-19 pandemic, with peaks precisely following the WHO's various announcements (e.g., public health emergency of international concern and mask-wearing in public areas). Opposing some beliefs, the burden of COVID-19 has not been dissuading anti-vaccine searches (23).

The COVID-19 pandemic has also changed parents' intentions to vaccinate their children. In the group of previously hesitant parents about vaccines, 50.9% were still reluctant during the pandemic, 40.0% of them consider that vaccines are necessary, and 9.1% were uncertain (24). Goldman et al. (25) reported that parents' intentions to vaccinate their children against influenza increased by 15.8% during the COVID-19 pandemic compared to the previous year (25).

Lassi et al. (26) reported a decline in the number of vaccines administered and vaccine coverage, which led to the rise of polio cases ~4 times in polio-endemic countries. Moreover, measles vaccination has been postponed in 23 countries. Even with a slight decrease in measles immunisation, it could lead to large outbreaks, considering the highly infectious nature of measles. Low vaccine coverage is influenced by factors, such as restriction on city-wide movement or fear of being exposed to the virus at healthcare facilities (26).

## Discussion

Trends on COVID-19 vaccine acceptance have modified as a result of an array of factors, decreasing between January 2020 and August 2021. Some countries are more receptive to vaccinate against COVID-19 than others, implying that context influences confidence and trust in vaccination. However, vaccine hesitancy is present across countries and subgroups, including healthcare professionals and parents (11). Parents argue that children are at lower risk of COVID-19, reflecting lower vaccine acceptance levels for children. While evidence points toward lower COVID-19 mortality rates in children, they still account for a sizeable proportion of COVID-19 cases (27). In fact, according to a preprint containing data from the UK, children and adults under 50 may be up to 2.5 times more likely to be infected with the surging Delta variant (28). Data from Taiwan further shows that ~50% of children infected with COVID-19 still retain symptoms half a year after (29). As such, childhood vaccination is increasingly called for and deployed as Delta surges (27, 30, 31). Nonetheless, most parents and guardians reported they would either accept a COVID-19 vaccine for themselves and their children or that they were unsure but leaning toward acceptance (12).

In the UK, negative news about the AstraZeneca vaccine did not appear to diminish UK citizens' trust in this vaccine. However, the study presents a limited sample, resulting in the bias of a non-representative sample (17). Therefore, further studies which include other settings may be beneficial to obtain more accurate conclusions.

Healthcare professionals play a key role in vaccination efforts (11), particularly as they are regarded as the most trusted source of information to inform patients and the public about vaccines. Healthcare professionals capable of understanding potentially hesitant patients can also appropriately respond to safety concerns, and best explain the benefits of vaccination. Greek healthcare professionals demonstrates a high vaccine acceptance rate for COVID-19 (80%) among physicians, which is higher than a previous study conducted in the same region at the beginning of the COVID-19 pandemic (13). The main reason for vaccine refusal was fear of potential side effects, which is in line with general population vaccine hesitancy.

Fear of side effects, safety, and effectiveness are the most common concerns about new vaccines, primarily because of the rapid development of COVID-19 vaccines. These fears are mainly contingent on social media misinformation, distrust in health authorities, and conspiracy theories. Accurate and scientific information is crucial to counterbalance numerous non-factual data shared in the media and social media platforms (14). Reliable sources with accurate information about vaccines, including adapted language to achieve the general population, should constitute a priority of Ministries of Health and healthcare professionals, with the primary goal to increase vaccine uptake and acceptance, particularly for COVID-19. Globally, social media platforms are becoming increasingly popular, leading to a growing number of public health concerns regarding the impacts of anti-vaccination content. Consequently, this constitutes a threat to emerging vaccines' receptivity. Forthcoming work on addressing this issue should promote evidence-based health literacy (21). People need to be informed correctly by the national public health authorities about the efficacy and safety of vaccines to promote confidence in vaccines and increase vaccination coverage (24).

The factors mentioned above influence not only COVID-19 vaccine hesitancy but also trust in other vaccines. The intention to get the COVID-19 and other vaccines are modified during the pandemic developments (24, 32), affecting routine immunisation. Vaccine hesitancy, reduction of vaccination coverage, and refusal to vaccinate during this pandemic may adversely impact the uptake of COVID-19 vaccines and the “regular” vaccines, such as vaccines against measles and polio (26). However, we have to acknowledge that the results of this mini review primarily covers the first phase of the pandemic. The substantial developments and official approval in the later stages of 2021 may have changed the perspectives covered in this study.

## Conclusions

Vaccine hesitancy constitutes a threat to tackling the COVID-19 pandemic because herd immunity depends on both the availability of vaccines and the population's willingness to accept those vaccines. Several concerns about vaccines are still arising, and diverse factors influence people's beliefs on vaccination, not only in the new vaccines but also in routine immunisation. Media and social media are vehicles of false news and non-factual information about vaccines in general. The general population needs appropriate, accurate, and high-quality information and, for this reason, more reliable sources with accurate and scientific information that is easily digestible by the wider population should be a priority of governments. Additionally, several efforts by the national public health authorities should be undertaken to improve vaccine acceptance and coverage. Further research may provide a better understanding of the effects of COVID-19 on vaccine hesitancy to determine future action on decreasing this problem.

## Author Contributions

MA: conceptualisation, methodology, data collection, data analysis, writing—original draft, and writing—review and editing. BR and BW: data analysis and writing—review and editing. RK: conceptualisation, methodology, writing—original draft, writing—review and editing, and supervision. EM: writing—review and editing, funding. All authors contributed to the article and approved the submitted version.

## Funding

BR was supported by Merck Sharp & Dohme (MSD).

## Author Disclaimer

The views and opinions expressed in this article are the authors' own and do not represent the institution they represent.

## Conflict of Interest

BR participated in this research independently of her role at MSD, where she is an employee. Furthermore, MSD did not provide funding or other support for this research. The authors declare that the research was conducted in the absence of any commercial or financial relationships that could be construed as a potential conflict of interest.

## Publisher's Note

All claims expressed in this article are solely those of the authors and do not necessarily represent those of their affiliated organizations, or those of the publisher, the editors and the reviewers. Any product that may be evaluated in this article, or claim that may be made by its manufacturer, is not guaranteed or endorsed by the publisher.
