# Supplementary material for: The Relationship Between the COVID-19 Pandemic and Vaccine Hesitancy: A Scoping Review of Literature Until August 2021
Source: Front Public Health. 2021 Sep 28;9:747787. doi: 10.3389/fpubh.2021.747787 (PMC8505886; doi:10.3389/fpubh.2021.747787)
Supplement: Supplementary file 1 [file Data_Sheet_1.docx]

de Albuquerque Veloso Machado M, Roberts B, Wong BLH, van Kessel R, and Mossialos E. The Relationship Between the COVID-19 Pandemic and Vaccine Hesitancy: A Scoping Review of Literature until August 2021.

**eMethods.** Details on the rationale and methodology of the scoping review.

**eTable 1**. Search terms used in combination in PubMed and Web of Science.

**eTable 2**. Inclusion and exclusion criteria

**eReferences.** Supplementary reference list.

**eMethods.** Details on the rationale and methodology of the scoping review.

**Research type and design**

A scoping review is particularly useful when a body of literature has a complex and heterogeneous nature and is previously under-researched [1,2]. It is also widely used to investigate emerging fields in order to assess the extent, type, and nature of the evidence that is available. The development of vaccine hesitancy during the COVID-19 pandemic is one of these emerging fields, making a scoping review a well-suited methodology to explore this emerging field and summarize and disseminate research findings, as well as to identify action points for future work [1,2].

**Data Sources and searches**

A search was performed between 6 and 11 April 2021. The databases used to perform data collection were PubMed and Web of Science. Given the scope of this review, four components were used to frame the search terms for the identification of studies: (1) COVID-19, (2) vaccines, (3) confidence, (4) impact. As presented in Table 1, several forms of the previous words, as well as relevant synonyms, were considered. In this search, MeSH terms were identified and included to ensure uniformity and consistency in the literature research.

**Study selection**

Various inclusion and exclusion criteria were applied in the study selection. Table 2 presents the eligibility criteria of this review. Articles published between 2020 and 2021 were included in this review, since the COVID-19 vaccines started to be developed in 2020. Editorials and commentaries were additionally included because of the newness of the topic, where experts’ opinion may be valuable for the findings. Small/specific study populations were excluded from this review because vaccine hesitancy is influenced by the context and data from very specific settings do not permit to formulate global conclusions on the topic.

**Data extraction and analysis**

After the use of the above-mentioned databases and the combination of search terms presented in eTable 1, all of the articles were added to Mendeley to remove the duplicates. The first screening of the publications collected in PubMed and Web of Science was based on the title and abstract of the articles. At this stage, publications were considered potentially relevant if their title and abstract demonstrate a link with the topic, following the eligibility criteria. For the second screening, the studies were downloaded, and the text was fully screened. Publications that fit the inclusion criteria defined in eTable 2 were classified as relevant and selected for the review.

**Study limitations**

This review is subject to limitations. The fact that this scoping review was developed in a short period, can present selection bias. Moreover, since this is a hot topic at this moment, the information presented in this review is contemporary and subject to change. The COVID-19 pandemic and vaccines are a new matter, which constitutes a limitation to this study, since there are a small number of studies and comparative results cannot be presented yet. Every day, new developments are being made related to the new vaccines. Furthermore, negative news about the side effects of the vaccines are emerging daily, influencing the confidence in vaccination all over the world. For further research on the topic, more studies should be conducted to develop conclusions about how these news influence public opinion, to address them in the proper manner.

**eTable 1**. Search terms used in combination in PubMed and Web of Science.

| 1 | AND | 2 | AND | 3 | AND | 4 |
| --- | --- | --- | --- | --- | --- | --- |
| COVID-19*  OR  SARS-COV-2* |  | COVID-19 Vaccine*  **OR**  Vaccines*  **OR**  Vaccination* |  | Acceptance  **OR**  Confidence  **OR**  Distrust  **OR**  Fear  **OR**  Hesitancy  **OR**  Mistrust  **OR**  Skepticism  **OR**  Trust*  **OR**  Uncertainty  **OR**  Uptake |  | Consequences  **OR**  Effect  **OR**  Impact  **OR**  Influence |

*MeSH term

**eTable 2**. Inclusion and exclusion criteria

| **Criteria** | **Inclusion** | **Exclusion** |
| --- | --- | --- |
| **Date and language** | Articles published between 2020 and 2021; Studies in English; Full paper available | Studies not in English language; Limited access |
| **Methodology** | Observational studies; Systematic Reviews; Rapid Reviews; Commentaries and Editorials | Cost-effectiveness studies; Randomized Controlled Trials; Mathematical modeling; Estimations |
| **Context** | General population; Healthcare workers; COVID-19 pandemic | Small/specific study populations, such as Amish, Roma and university students in a certain country. |
| **Outcomes** | Studies that include: (1) Trends on COVID-19 vaccine hesitancy; (2) COVID-19 pandemic influence on healthcare workers’ vaccine uptake; (3) Factors that influence vaccine acceptance in COVID-19 era (4) Impact of COVID-19 pandemic on vaccine hesitancy | Studies including: Biomedical evaluation of COVID-19 vaccines; Management of COVID-19 pandemic; Mental Health effects of COVID-19; Ethical aspects of COVID-19 vaccines |

**eReferences.** Supplementary reference list.

1. Levac D, Colquhoun H, O’Brien KK. Scoping studies: advancing the methodology. Implement Sci. 2010 Dec 20 [cited 2018 Apr 25];5(1):69. Available from: http://www.ncbi.nlm.nih.gov/pubmed/20854677

2. Arksey H, O’Malley L. Scoping studies: towards a methodological framework. Int J Soc Res Methodol. 2005 Feb [cited 2018 Apr 25];8(1):19–32. Available from: http://www.tandfonline.com/doi/abs/10.1080/1364557032000119616
